# Targeted Disruption of Ephrin B1 in Cells of Myeloid Lineage Increases Osteoclast Differentiation and Bone Resorption in Mice

**DOI:** 10.1371/journal.pone.0032887

**Published:** 2012-03-05

**Authors:** Shaohong Cheng, Shien Lucy Zhao, Brittany Nelson, Chandrasekhar Kesavan, Xuezhong Qin, Jon Wergedal, Subburaman Mohan, Weirong Xing

**Affiliations:** 1 Musculoskeletal Disease Center, Jerry L Pettis VA Medical Center, Loma Linda, California, United States of America; 2 Department of Medicine, Loma Linda University, Loma Linda, California, United States of America; 3 Department of Biochemistry, Loma Linda University, Loma Linda, California, United States of America; 4 Department of Physiology, Loma Linda University, Loma Linda, California, United States of America; University of Maryland School of Medicine, United States of America

## Abstract

Disruption of ephrin B1 in collagen I producing cells in mice results in severe skull defects and reduced bone formation. Because ephrin B1 is also expressed during osteoclast differentiation and because little is known on the role of ephrin B1 reverse signaling in bone resorption, we examined the bone phenotypes in ephrin B1 conditional knockout mice, and studied the function of ephrin B1 reverse signaling on osteoclast differentiation and resorptive activity. Targeted deletion of ephrin B1 gene in myeloid lineage cells resulted in reduced trabecular bone volume, trabecular number and trabecular thickness caused by increased TRAP positive osteoclasts and bone resorption. Histomorphometric analyses found bone formation parameters were not changed in ephrin B1 knockout mice. Treatment of wild-type precursors with clustered soluble EphB2-Fc inhibited RANKL induced formation of multinucleated osteoclasts, and bone resorption pits. The same treatment of ephrin B1 deficient precursors had little effect on osteoclast differentiation and pit formation. Similarly, activation of ephrin B1 reverse signaling by EphB2-Fc treatment led to inhibition of TRAP, cathepsin K and NFATc1 mRNA expression in osteoclasts derived from wild-type mice but not conditional knockout mice. Immunoprecipitation with NHERF1 antibody revealed ephrin B1 interacted with NHERF1 in differentiated osteoclasts. Treatment of osteoclasts with exogenous EphB2-Fc resulted in reduced phosphorylation of ezrin/radixin/moesin. We conclude that myeloid lineage produced ephrin B1 is a negative regulator of bone resorption *in vivo*, and that activation of ephrin B1 reverse signaling inhibits osteoclast differentiation *in vitro* in part via a mechanism that involves inhibition of NFATc1 expression and modulation of phosphorylation status of ezrin/radixin/moesin.

## Introduction

Osteoporosis is an aging-related major health problem in women and men. There are two major known causes of osteoporosis; low peak bone mineral density that is typically achieved around the age of 30, and high bone loss rate which occurs particularly after menopause and during the natural process of aging. Bone loss occurs with age in part because the increased bone resorption rate is not compensated for by the corresponding increase in the bone formation rate. Therefore, studies to identify the regulatory factors and their molecular pathways that modulate bone resorption rate are important to the overall understanding of bone diseases.

Ephrin (A and B) and their receptors have been shown to play key roles in the growth and development of multiple tissues [1], [2], [3]. Ephrin As are membrane anchored proteins while ephrin Bs are transmembrane proteins. In general, ephrin As bind to eprhin A receptors while ephrin Bs bind to ephrin B receptors (EphBs) with few exceptions [4]. The interaction of ephrin B with its receptors via cell-cell contact leads to the activation of a bidirectional signal in which both the receptors (forward) and the ligand (reverse) activate downstream signaling cascades [5], [6], [7], [8]. Ephrin B1, B2, and B3 have the same structure of a single transmembrane domain, and a well-conserved cytoplasmic domain that includes 33 amino acids with 100% identity [9], [10]. Studies in non-bone cells have shown that the PDZ binding motif, and six tyrosine residues within the C-terminal 33 amino acids of ephrin B1 and B2 function as receptor-like signaling molecules which transduce signals into the interior of the cell through tyrosine phosphorylation and interaction with PDZ domain-containing proteins [11], [12], [13], [14]. In addition to tyrosine phosphorylation, there is also evidence for phosphorylation of serine residues in ephrin B1 by serine/threonine kinases to facilitate binding of adapter proteins [15]. In our previous studies, we have shown that sodium hydrogen exchange regulatory factor 1 (NHERF1) interacts with ephrin B1, recruits other PDZ proteins and mediates transcription factor TAZ dephosphorylation and nuclear transportation leading to increased expression of genes that are critical for osteoblast differentiation [16].

Both ephrin B ligands and their receptors are co-expressed in osteoblasts, but only ephrin B1 and B2 are expressed during the osteoclast precursor differentiation [7]. While *in vitro* studies have shown that activation of ephrin B2 in osteoclasts inhibited c-Fos and NFATc1 expression, leading to decreased osteoclast differentiation, specific disruption of ephrin B2 in myeloid lineage cells did not induce bone resorption or cause bone loss *in vivo* [7]. The lack of skeletal phenotype in ephrin B2 conditional knockout (KO) mice could be due to a functional compensation by ephrin B1 since the C-terminal domains of ephrin B1 and ephrin B2 are structurally similar. Since little is known on the role of ephrin B1 in regulating osteoclast functions, we examined if activation/inactivation of ephrin B1 reverse signaling modulates macrophage colony-stimulating factor (M-CSF)/receptor activator of nuclear factor κβ ligand (RANKL) signaling and alters mature osteoclast formation *in vivo* and *in vitro*. Our studies found that ephrin B1 was expressed at a several-fold higher level than ephrin B2 during osteoclast differentiation, and mice with conditional disruption of ephrin B1 gene in cells of myeloid lineage had reduced trabecular bone volume, trabecular number, trabecular thickness, and increased trabecular separation.

## Results

### Ephrin B1 is predominantly expressed in osteoclasts

**Figure 1:**
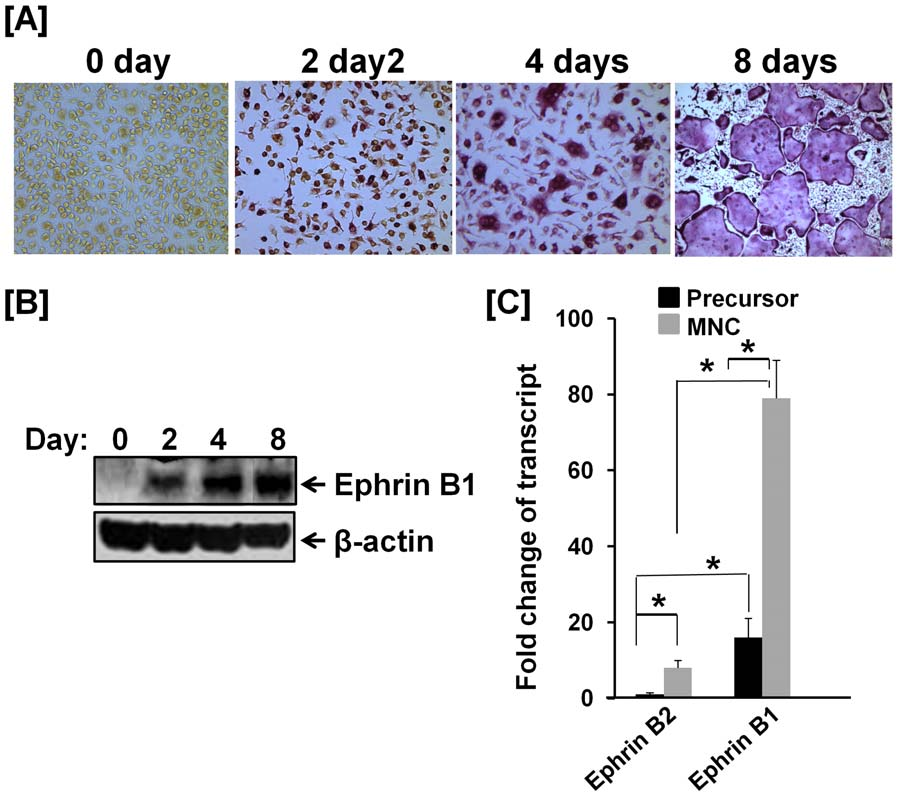
Expression of ephrin B1 is increased during osteoclast differentiation. [**A–B**]: Expression of ephrin B1 is increased during osteoclast differentiation. Osteoclast precursors isolated from non-adherent bone marrow of wild-type (WT) mice were treated with 20 ng/ml of M-CSF and 30 ng/ml of RANKL for 0, 2, 4 and 8 days. The differentiated multinuclear cells (MNCs) were monitored by TRAP staining. The parallel cultures were harvested, and total cellular proteins were extracted for Western blot. [**C**]: Expression of ephrin B1 is more abundant than ephrin B2 in osteoclasts. Mouse splenocytes were treated with M-CSF only or M-CSF plus RANKL for 4 days. Total RNA was extracted for real-time PCR. Values are fold-change over the expression level of ephrin B2 transcript in undifferentiated precursors, and expressed as mean ± SEM (standard error of mean) (n = 3). A star represents statistical significance of expression level of transcript in MNCs as compared to the precursors or between two groups indicated (P<0.01).

Because both ephrin B1 and ephrin B2 were reported to be expressed in osteoclasts [7], we compared protein expression levels of ephrin B1 and ephrin B2 during the differentiation of precursors to mature multinucleated osteoclasts. Osteoclast precursors derived from the spleen or non-adherent bone marrow of wild-type (WT) mice were cultured in the presence of 20 ng/ml of M-CSF and 30 ng/ml of soluble RANKL for 8 days. Precursors cultured in the absence of soluble RANKL serve as undifferentiated controls (0 day). As shown in Figure 1A, precursors derived from non-adherent bone marrow were differentiated to tartrate-resistant acid phosphatase (TRAP) staining positive, multinucleated cells (MNC) after 4–8 days of RANKL treatment. A parallel set of undifferentiated and differentiated osteoclasts were lysed for Western blot with specific antibodies to mouse ephrin B1, ephrin B2, and β-actin (Figure 1B). The expression levels of ephrin B1 protein continued to increase during the differentiation of osteoclast precursors into mature osteoclasts. The highest expression of ephrin B1 was found in mature MNCs at day 8 after RANKL treatment. However, the expression of ephrin B2 protein was undetectable when 30 µg of total cellular protein extracted from the differentiated and undifferentiated osteoclasts was analyzed by Western blot using a commercial polyclonal antibody which did recognize recombinant ephrin B2 protein over-expressed in RAW264.7 positive control cells (data not shown). To quantify the expression levels of ephrin B1 and ephrin B2 transcripts during the osteoclast differentiation, we isolated precursors from mouse spleen, and treated them with M-CSF and RANKL for 4 days. The cells were then lysed for RNA extraction and real time RT-PCR. While expression of both ephrin B1 and ephrin B2 was significantly increased in differentiated MNCs as compared to undifferentiated cells, expression of ephrin B1 was 15-fold higher than ephrin B2 in undifferentiated precursors after calibration of PCR amplification efficiencies for ephrin B1 primers and ephrin B2 primers with cDNA containing plasmid. The expression level of ephrin B1 was 10 times higher than ephrin B2 in the mature MNCs. Ephrin B2 transcript was increased by 8-fold in the MNCs while the expression of ephrin B1 was elevated by 5.3 fold in the differentiated osteoclasts as compared to the undifferentiated precursors (Figure 1C).

### Activation of ephrin B1 reverse signaling inhibits osteoclast differentiation

**Figure 2:**
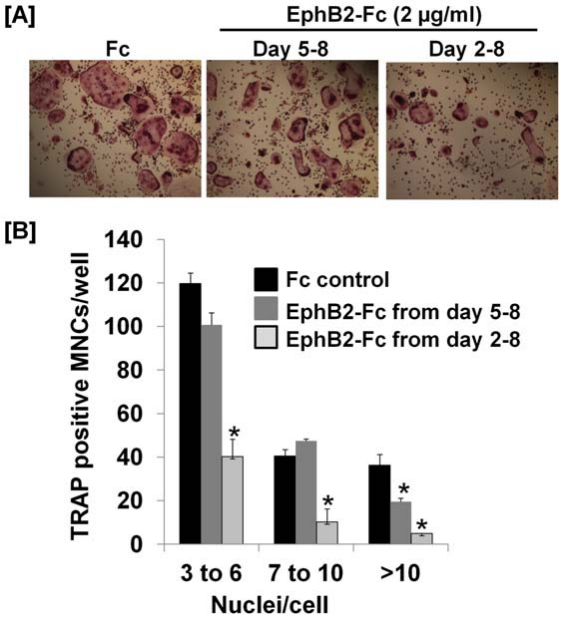
Activation of ephrin B1 reverse signaling suppresses osteoclast differentiation. [**A**]: Osteoclast precursors derived from WT mice were differentiated in the presence of 20 ng/ml of M-CSF and 30 ng/ml of RANKL for 8 days. The cells were also treated with clustered EphB2-Fc during day 2–8 or day 5–8 of the culture as indicated in the figure, followed by TRAP staining. [**B**]: Quantitative data of TRAP positive MNCs. Values are mean ± SEM (n = 8). A star presents statistical significance of MNCs in the differentiated cultures treated with EphB2-Fc as compared to the cultures treated with control Fc (P<0.01).

To determine if activation of ephrin B1 reverse signaling influences the differentiation of osteoclast precursors into mature MNCs, bone marrow macrophage precursors were treated with clustered soluble recombinant EphB2-Fc protein that only contains extracellular domain of mouse EphB2 receptor, and can activate ephrin B1 reverse signaling but not EphB2 mediated forward signaling during day 2–8 or day 5–8 of RANKL-induced osteoclast differentiation. As shown in Figure 2, eight days of RANKL treatment induced formation of mature MNCs from the precursors derived from the WT mice that was inhibited by exogenous addition of EphB2-Fc. Treatment of osteoclasts with EphB2-Fc from day 2–8 resulted in greater inhibition of osteoclast formation than day 5–8. Formation of multinucleated cells with 3–6, 6–10 and >10 nuclei per cell were reduced by 60%, 80% and 95%, respectively, in bone marrow macrophage precursors treated with EphB2-Fc during day 2–8 as compared to control Fc. However, treatment of osteoclast precursors during days 5–8 with EphB2-Fc caused no significant difference in the formation of differentiated osteoclasts with 3–10 nuclei while it did decrease formation of large osteoclasts with more than 10 nuclei per cell by 50%.

### Disruption of ephrin B1 gene in cells of myeloid lineage reduced trabecular bone volume, trabecular number and bone thickness

**Figure 3:**
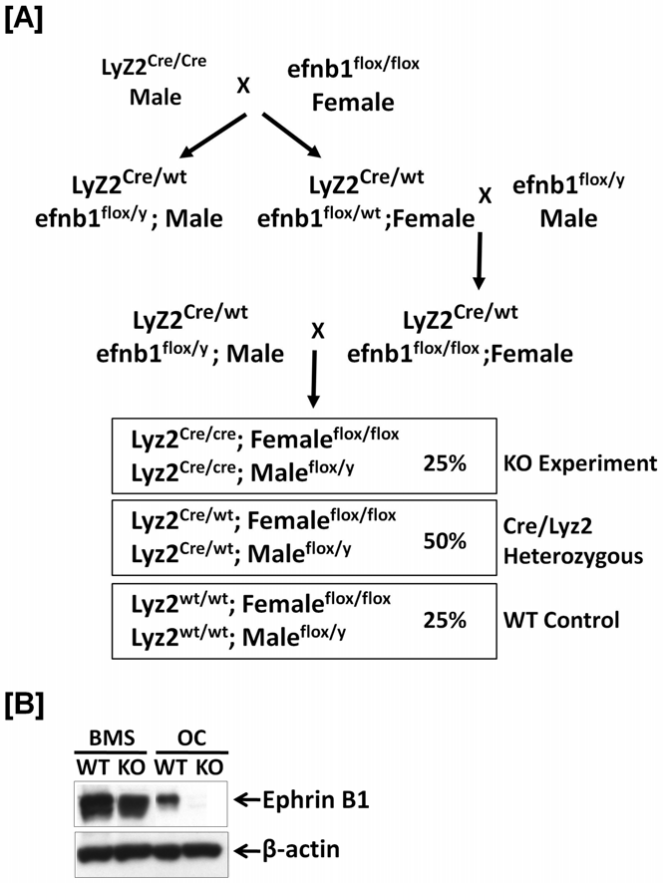
Conditional ephrin B1 KO is myeloid-lineage specific. [**A**]: Schematic diagram of generation of ephrin B1 conditional KO and control WT. Mice with ephrin B1 deletion in myeloid lineage cells are generated by crossing ephrin B1 loxp mice with Lyz2-Cre knock-in mice. [**B**]: Ephrin B1 is not expressed in osteoclasts from the myeloid-specific conditional KO mice. Splenocytes derived from WT and KO mice were cultured in the presence of 20 ng/ml of M-CSF and 30 ng/ml of RANKL for 3 days. Bone marrow stromal (BMS) cells isolated from the long bones were also cultured in α-MEM medium containing 10% FBS for 6 days. Osteoclasts (OC) and BMS cells were harvested, respectively, and the cellular proteins were extracted for measurement of ephrin B1 protein, by Western blot.

**Figure 4:**
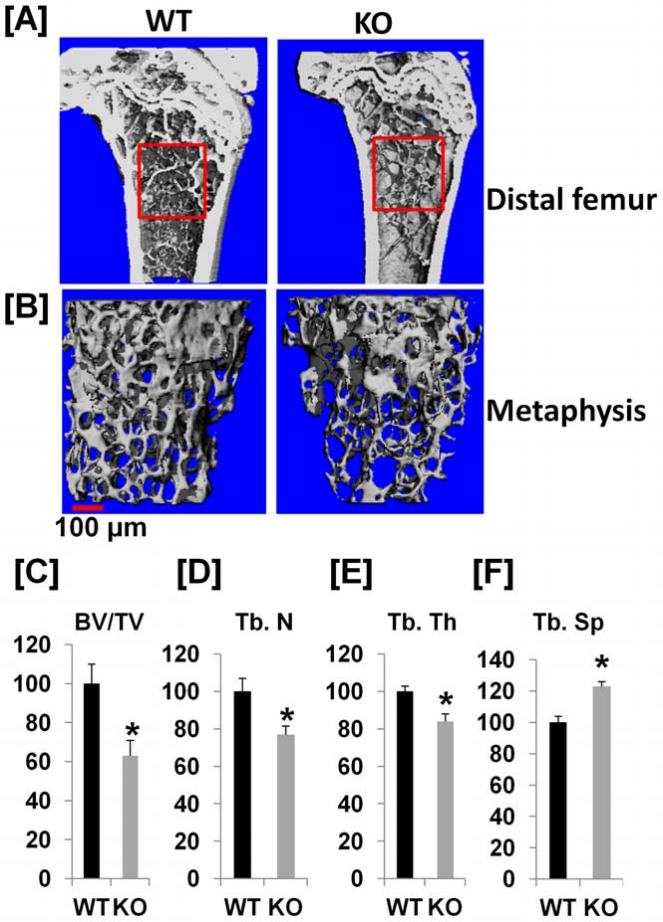
Ephrin B1 deletion in cells of myeloid lineage decreases trabecular bone. [**A**]: Longitudinal section of μ-CT images of distal femurs of WT and KO mice. The metaphysis of distal femurs were selected for analyses of trabecular bone parameters by μ-CT. [**B**]: μ-CT images of trabecular bone of the distal metaphysis of the femurs. [**C–F**]: Quantitative measurements of trabecular bone at the metaphysis of distal femurs. [**C**]: Percentage change of trabecular bone volume/total bone volume (BV/TV) of the distal femur of KO mice as compared to WT littermate controls. [**D**]: Percentage change of trabecular number (Tb. N) of the distal femur of KO mice as compared to WT littermate controls. [**E**]: Percentage change of trabecular thickness (Tb. Th) of the distal femur of KO mice as compared to WT littermate controls. [**F**]: Percentage change of trabecular separation (Tb. Sp) of the distal femur of KO mice as compared to WT littermate controls. Values are expressed as mean ± SEM (n = 8). A star presents statistical significance (P<0.05) as compared to WT littermate controls.

To study the functions of ephrin B1 expressed in osteoclasts, ephrin B1 gene was deleted in cells of myeloid lineage by Cre/loxp approach (Figure 3A). After 3 generations of breeding, homozygous Lyz2-Cre, loxp homozygous female or hemizygous male mice were generated, and used as experimental KO mice. Both WT alleles of Lyz2 gene, loxp homozygous or hemizygous mice served as controls. To confirm the ephrin B1 expression in the homozygous Lyz2-Cre, loxp homozygous mice, precursors were isolated from the spleen of conditional KO and WT mice, and differentiation was induced by treatments of M-CSF and RANKL. Total cellular extracts from differentiated osteoclasts were used for Western blot. As expected, the expression of the ephrin B1 protein was absent in osteoclasts from homozygous Lyz2-Cre, loxp homozygous mice, but was detected in the cells from Cre- control littermates (Figure 3B). In contrast, bone marrow stromal cells derived from both KO and WT mice expressed high levels of ephrin B1 protein. To characterize the skeletal phenotypes of ephrin B1 conditional KO mice, the femurs were collected from 21 week old mice and analyzed trabecular structures by μ-CT analyses (Figures 4A–B). While there was no significant difference in cortical mineral density at the mid diaphysis of the femur of WT and KO mice (data not shown), the ratio of trabecular bone volume to total volume (BV/TV) was reduced by 40% at the metaphysis of the femur from the mixed genders of KO mice as compared to the littermate controls (Figure 4C). Trabecular number and trabecular thickness were reduced by 23% and 18%, respectively, while trabecular separation was increased by 24% at this site of the femurs isolated from KO mice (Figures 4 D–F). There was no significant difference in the magnitude of trabecular bone volume reduction in the KO mice between the two genders (data not shown).

**Figure 5:**
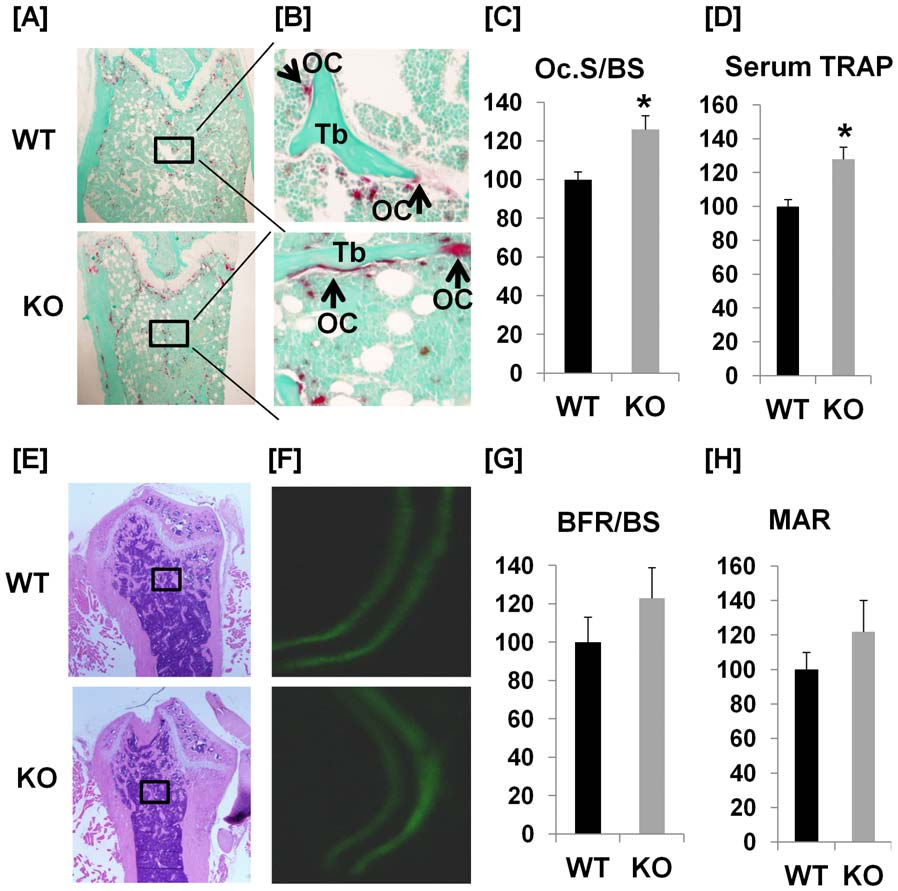
Ephrin B1 deletion in cells of myeloid lineage increases bone resorption. [**A–B**]: TRAP staining of osteoclasts at trabecular surface of metaphysis of the femur in WT and KO mice at 21 weeks of age. Bone section was counterstained with methyl green. Arrows denote representative TRAP positive osteoclasts. A: 20× magnification, B: 100× magnification. [**C**]: Percentage change of TRAP labeled surface/trabecular bone surface (Oc. S/BS) in the bone of ephrin B1 KO mice as compared to WT controls at 21 weeks of age. Values are presented as mean ± SEM (n = 8). [**D**]: Percentage change of serum TRAP activity of ephrin B1 KO mice as compared to WT controls at 21 weeks of age. Values are presented as mean ± SEM (n = 8). [**E–F**]: Trabecular bone formation is unaffected in mice with deletion of ephrin B1 in myeloid lineage cells. E: Images of H & E staining of distal femurs of WT and KO mice (20×). F: representative images of calcein double labeled trabecular bone of WT and KO mice (200×). [**G–H**]: Quantitative data of trabecular bone formation measured at distal femur metaphysis of WT and KO mice at 21 weeks of age. G: Bone formation rate/bone surface (BFR/BS). Values are presented as mean ± SEM (n = 8). H: Mineral apposition rate (MAR). Values are presented as mean ± SEM (n = 8).

To identify the target cell types and cellular processes that contribute to reduced trabecular bone volume of the distal femurs in the ephrin B1 conditional KO mice, histomorphometric studies were performed in KO and control mice. Figure 5 shows the data from TRAP staining for trabecular bone surfaces examined at distal metaphysis of the femurs of mixed genders of 21 week old mice (N = 8 with 50% females and 50% males). Two longitudinal sections from the middle sampling site of the femur were TRAP-stained. Total trabecular surface and TRAP positive tabecular surface in 7 microscope fields per section were blindly quantified with OsteoMeasure software. We found that the percentage of TRAP labeled surface to bone surface at the metaphysis was increased by 26% in the conditional KO mice as compared to the littermate controls (Figures 5A–C). In addition, the serum TRAP activity, a bone resorption marker, was also elevated by 28% in the KO mice (Figure 5D). We next determined if loss of ephrin B1 in cells of myeloid lineage influences bone formation. We found there was no significant difference in newly formed bone between two calcein labels in the KO mice as compared to control mice (Figures 5E & F). The bone size was not affected in the KO mice (data not shown). Bone formation rate/bone surface (BFR/BS) and mineral apposition rate (MAR) were not significantly altered (Figures 5G & H). The lack of difference in bone formation between conditional KO and WT mice was not surprising because conditional disruption of ephrin B1 in cells of myeloid lineage did not influence expression of ephrin B1 in bone marrow stromal cells as shown by Western blot analyses (Figure 3B).

### Activation of ephrin B1 reverse signaling inhibits osteoclast differentiation and bone resorption

**Figure 6:**
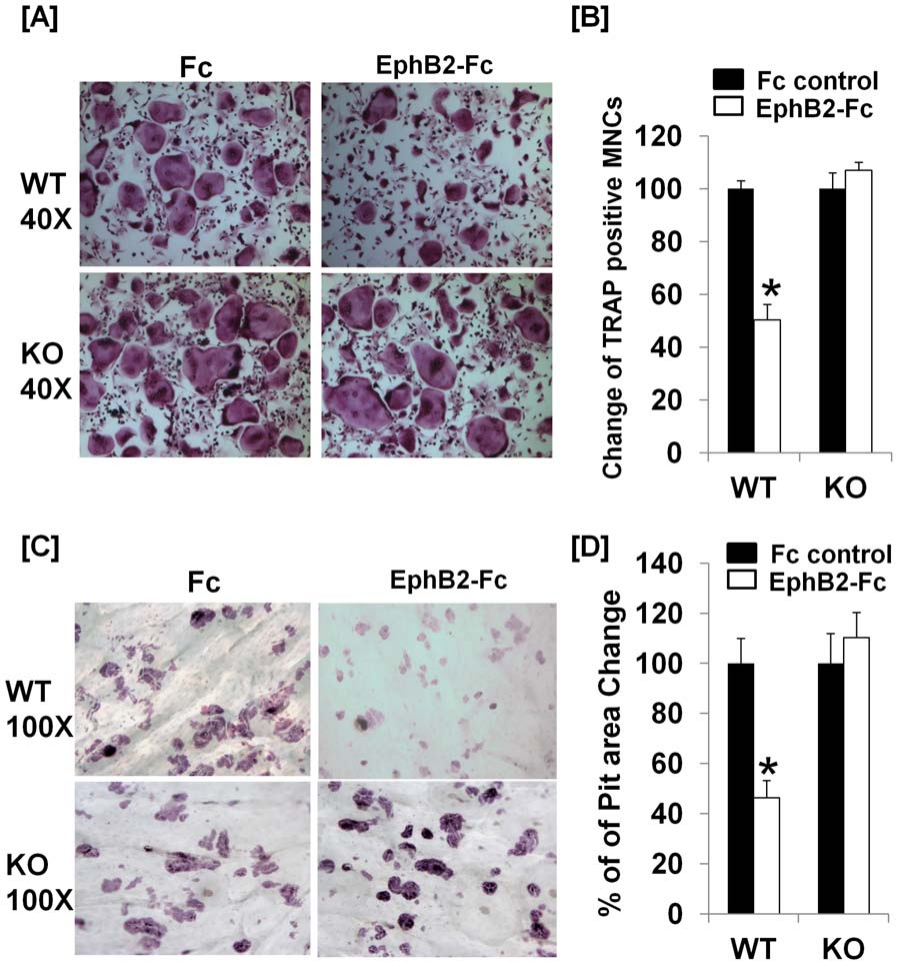
Activation of ephrin B1 reverse signaling inhibits osteoclast formation and bone resorption. [**A**]: Interaction of ephrin B1 with EphB2-Fc suppresses osteoclast differentiation. Splenocytes from WT and ephrin B1 KO mice were cultured in the presence of RANKL and M-CSF for 24 hours, and then treated with soluble EphB2-Fc (2 µg/ml) or control Fc for 8 days. TRAP staining was performed after 6 days EphB2-Fc treatment. MNCs were visualized under microscope. [**B**]: Quantification of MNCs (3 or more nuclei per cell). Values are mean ± SEM (n = 8). A star presents statistical significance (P<0.01) as compared to the cells treated with control Fc. [**C**]: Interaction of ephrin B1 with EphB2-Fc suppresses bone resorption. Splenocytes from WT and ephrin B1 KO mice were seeded on bone slices, and cultured in the presence of RANKL and M-CSF for 24 hours, and then treated with EphB2-Fc or control Fc for additional 8 days. Resorption pits on bone slices were analyzed after 8 days of EphB2-Fc treatment. [**D**]: Quantification of resorption pit areas. Values are mean ± SEM (n = 6). A star presents statistical significance (P<0.01) as compared to the cells treated with control Fc.

To examine whether lack of ephrin B1 expression and ephrin B1 reverse signaling influences osteoclast differentiation, splenocytes were isolated from WT and ephrin B1 conditional KO mice, and induced to differentiate in the presence of M-CSF and RANKL. Cells were also treated with EphB2-Fc or control Fc at day 2 through 10, followed by TRAP staining. Multinucleated cells were counted under the microscope. As shown in Figures 6A & B, treatment of WT precursors with EphB2-Fc suppressed MNCs formation by 49.8% while the same treatment of ephrin B1 deficient cells with EphB2-Fc had little effect on osteoclast differentiation as compared to Fc control treatment. To further test the effect of ephrin B1 reverse signaling on osteoclast function, bone resorption pit assay was performed. Osteoclast precursors were seeded on the top of bone slices in the presence of M-CSF and RANKL, and treated with EphB2-Fc or control Fc in the same way as described above. After 10 days of culture, bone resorption pits were stained and the resorption areas were analyzed. As shown in Figures 6C & D, treatment of WT osteoclast precursors with EphB2-Fc inhibited area of resorption pits by 54% as compared to the cells treated with control Fc. In addition, the size of individual resorption pits appeared smaller in the EphB2-Fc treated cultures as compared to Fc controls. However, the same treatment of ephrin B1 deficient cells did not show a significant effect on pit formation.

**Figure 7:**
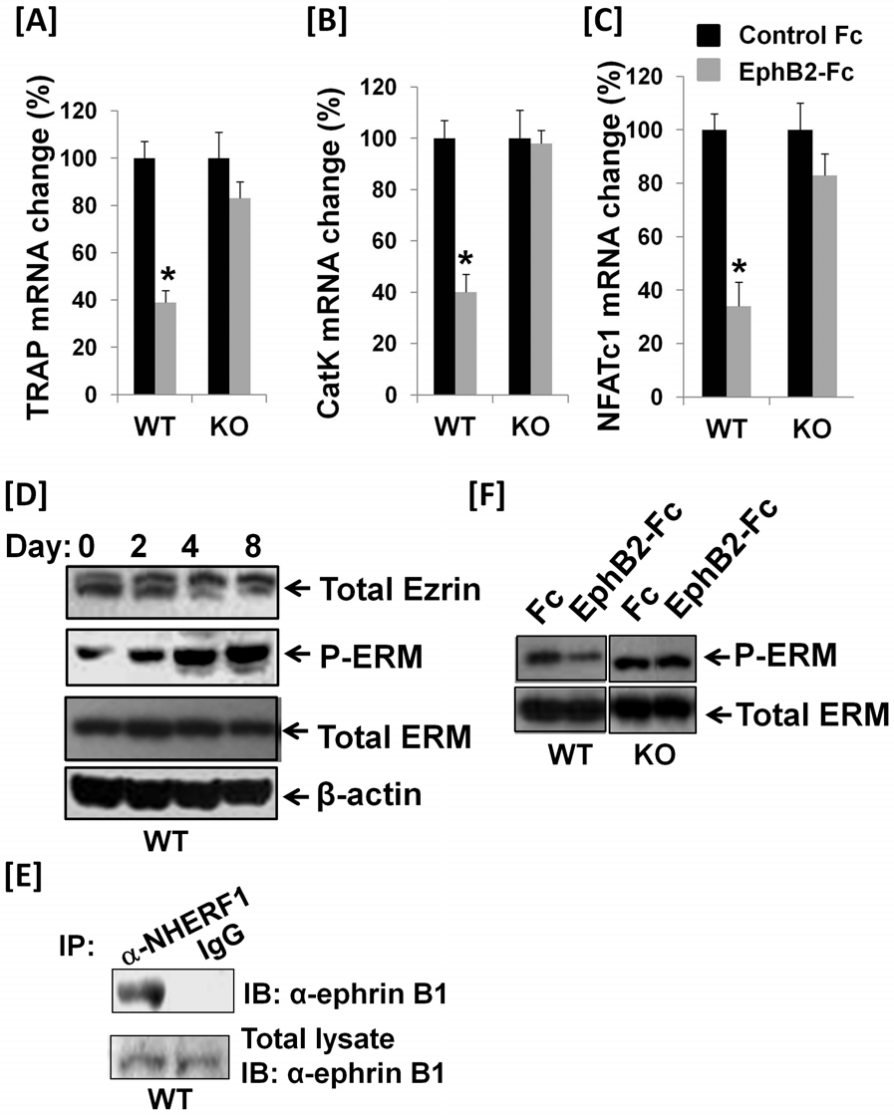
Activation of ephrin B1 reverse signaling inhibits phosphorylation of ezrin/radixin/moesin and RANKL target gene expression in osteoclasts. [**A–C**]: Treatment of EphB2-Fc inhibits expression of osteoclast differentiation marker genes. Precursors were cultured in the presence of RANKL and M-CSF for 24 hours, and then treated with soluble EphB2-Fc (2 µg/ml) or control Fc for another 4 days. Total RNA was extracted for real-time RT-PCR using specific primers to TRAP, cathepsin D (CatK) and NFATc1. Values are expressed as fold change over WT cells ± SEM (n = 3). A star presents statistical significance (P<0.05) as compared to the cells derived from WT littermate mice. [**D**]: ERM phosphorylation is increased during RANKL induced osteoclast differentiation. Splenocytes from WT mice were treated with 20 ng/ml of M-CSF and 30 ng/ml of RANKL for 0, 2, 4 and 8 days. Total cellular proteins from osteoclasts were extracted for Western blot. [**E**]: Ephrin B1 interacts with NHERF1 in osteoclasts. Splenocytes were treated with M-CSF and RANKL for 4 days. Cells were then treated with EphB2-Fc for 5 minutes, and used for immunoprecipitation. [**F**]: Activation of ephrin B1 reverse signaling inhibits ERM phosphorylation. Splenocytes derived from ephrin B1 KO and WT mice were treated with M-CSF and RANKL for 4 days, and then the cells were treated with 2 µg/ml of EphB2-Fc or Fc in differentiation medium for another 24 hours. Total cellular proteins were extracted for Western blot.

To examine whether activation of ephrin B1 reverse signaling influences expression of osteoclast differentiation marker genes, splenocytes derived from WT mice were cultured in the presence of RANKL and M-CSF for 24 hours, and then treated with soluble EphB2-Fc or control Fc for another 4 days. Total RNA was extracted for RT real-time PCR. As shown in Figure 7A, expression of TRAP gene was reduced by 62% in the differentiated osteoclasts in the EphB2-Fc treated cells as compared to the control cells treated with Fc, but there was no change in TRAP expression between the treatments in the undifferentiated precursors (data not shown). Similarly, EphB2-Fc treatment inhibited the expression of cathepsin K (CatK) and NFAFc1 by 60% and 67%, respectively, in WT osteoclasts, however not in the ephrin B1 deficient cells. There was little change in c-Fos expression in the WT cells treated with EphB2-Fc as compared to the cells treated with Fc (data not shown).

### Activation of ephrin B1 reverse signaling stimulates ephrin B1 interaction with NHERF1, and ezrin/radixin/moesin inactivation

Because the ezrin, radixin and moesin (ERM) proteins function as linkers between the plasma membrane and the actin cytoskeleton, and phosphorylation of ERM is involved in cytoskeletal rearrangement and cell migration [17], [18], [19], [20], and because NHERF1 expressed in osteoclasts associates with ezrin and actin [21], we predicted that activation of ephrin B1 reverse signaling would affect intra or intermolecular interaction between ERM amino- and carboxyl-terminal domains via complex formation of ephrin B1, NHERF1 and PDZ containing phosphatase. To test if ezrin expression and phosphorylation of ERM proteins were changed during osteoclast differentiation, total cellular proteins were extracted from different stages of osteoclast differentiation for immunoblot with specific antibodies against total ezrin, phosphor-ERM and total ERM. Western blot analyses with antibody against total ezrin detected two species of ezrin protein with molecular weights around 80 kDa. While the total ezrin was not changed during the differentiation of precursors into mature osteoclasts, larger molecular weight of ezrin proteins was increased at day 2, reached its peak at day 4 and maintained at highest level at day 8 in multinucleated cells (Figure 7D). In contrast, ezrin with smaller molecular mass was decreased in differentiated osteoclasts (days 4 & 8) as compared to the undifferentiated precursors (day 0). In addition, specific antibody against phosphorylated ERM at a carboxyl-terminal residue (threonine 567 of ezrin, threonine 564 of radixin, threonine 558 of moesin) detected elevated levels of active form of phosphorylated ERM in differentiated multinucleated cells as compared to the undifferentiated control cells (day 0).

To examine the interaction of ephrin B1 with NHERF1 in differentiated osteoclasts, we stimulated the cells with clustered EphB2-Fc for 5 min, cross-linked with dithiobis (succinimidyl) propionate, and lysed for immunoprecipitation with anti-NHERF1. As shown in Figure 7E, we were able to immunoprecipitate both ephrin B1 and NHERF1 in the lysate of differentiated osteoclasts. To further test if activation of ephrin B1 changes ERM phosphorylation at C-terminus of threonine residues in differentiated osteoclasts, osteoclast precursors were differentiated for 4 days, and then the differentiated cells were treated with 2 µg/ml of EphB2-Fc or Fc for 24 hours. Total cellular protein was extracted for Western blot with specific antibodies to total ERM and phosphorylated form of ERM (p-ERM). We found that activation of ephrin B1 by EphB2-Fc treatment inhibited ERM phosphorylation in the WT osteoclasts, but not in the ephrin B1 deficient cells (Figure 7F).

## Discussion

Ephrin B1 is expressed in various types of cells and plays key roles in the growth and development of multiple tissues [1], [2], [22], [23], [24], [25]. Although ubiquitous deficiency of ephrin B1 in every types of cells or in epiblast during early embryogenesis results in prenatal lethality and skeletal defects in mice [1], [5], and conditional KO of ephrin B1 gene in collagen I producing cells leads to calvarial defects and reduced bone formation [16], little is known on the role of ephrin B1 produced during osteoclast differentiation and maturation. In this study, we show that ephrin B1 was predominantly expressed in osteoclast precursors and differentiated osteoclasts. Ephrin B1 expression was increased at both mRNA and protein levels during the differentiation of precursors to multinucleated osteoclasts. We also used knock-in mice expressing a Cre recombinase under the control of endogenous Lyz2 regulatory elements to disrupt ephrin B1 in cells of myeloid lineage, and examined the consequence of conditional disruption of ephrin B1 on the skeletal phenotypes *in vivo*. We show that KO of ephrin B1 in osteoclast precursors was complete, and the conditional KO in myeloid lineage cells did not influence ephrin B1 expression in bone marrow stromal cells. Targeted disruption of ephrin B1 in cells of myeloid lineage resulted in decreased trabecular number, trabecular thickness, and trabecular bone volume but increased trabecular separation. The reduced trabecular bone in ephrin B1 conditional KO mice was caused by increase in osteoclast differentiation and bone resorption. Our *in vitro* studies also demonstrate that interaction of ephrin B1 with soluble EphB2-Fc suppressed ERM phosphorylation, osteoclast differentiation and resorption pit formation. The findings indicate that ephrin B1 predominantly produced in osteoclasts is an important regulator of osteoclast differentiation, bone resorption and trabecular bone volume.

Ephrin B1, B2 and B3 share the same structure of a single transmembrane domain, and a well-conserved cytoplasmic domain that includes 33 amino acids with 100% identity [9], [10]. Among ephrin B proteins, B1 and B2 have been shown to be expressed in bone cells [5], [7]. In terms of ephrin B1 and B2 functions, Zhao et al. generated conditional ephrin B2 KO mice by crossing ephrin B2 loxp mice with Lyz2-Cre, and examined the bone phenotypes *in vivo*. Although their studies demonstrated a critical role of ephrin B2 reverse signaling in osteoclast differentiation, mice with disruption of ephrin B2 in cells of myeloid lineage cells failed to show a significant increase in bone resorption [7]. This study suggested that other ephrin B ligands may compensate for the loss of ephrin B2 function in osteoclasts *in vivo*. In our studies, we found that while ephrin B2 protein levels were below detectable limit in both undifferentiated and differentiated osteoclasts in our culture condition, the expression of ephrin B1 protein was significantly elevated in osteoclast precursors, and was further increased during the osteoclast differentiation. Our result of Western blot was inconsistent with previous studies which demonstrate expression of ephrin B2 in differentiated osteoclasts [26]. One potential explanation for the discrepant results is that the amount of total cellular protein (30 µg) we used was not sufficient to detect low levels of endogenous ephrin B2 by Western blot with low affinity ephrin B2 antibody while the same dilution of antibody could detect the high concentration of over-expressed ephrin B2 protein in the cell extract from our culture system. Furthermore, loss of ephrin B1 did not cause up-regulation of ephrin B2 in osteoclast precursors. Accordingly, activation of ephrin B1 reverse signaling by EphB2-Fc treatment inhibited osteoclast differentiation in ephrin B1 producing WT cells, but not in ephrin B1 deficient cells. Our *in vitro* data together with *in vivo* data showing phenotypes of reduced trabecular bone volume in ephrin B1 conditional KO mice provide strong evidence that the amount of ephrin B2 protein in osteoclasts cannot compensate for the loss of ephrin B1 on the skeletal phenotype in the ephrin B1 conditional KO mice, and explain why mice with conditional KO of ephrin B2 in osteoclasts did not exhibit a bone phenotype.

In our previous studies, we have demonstrated that bone marrow stromal cells and calvarial osteoblasts express both ephrin B1 and its cognate EphB2 receptor [16]. Besides EphB2, ephrin B1 also preferentially binds B3 receptor with high affinity and interacts with EphB1, B4 and A4 receptors with low affinity [4], [7], [27], [28]. In the cells that co-express both ephrin ligand and its receptors, the ephrin ligand and receptor proteins can be segregated into distinct membrane domains from which they signal biological effects via cell surface interactions [6]. Thus, both forward and reverse signals are feasible upon contact of osteoclast to osteoblast or mesenchymal stem cell on the bone surface. In this regard, recent studies found that ephrin B2 produced by osteoclasts might interact with EphB4 receptor in osteoblasts to induce forward signaling and osteoblast differentiation [7], and EphB2 mediated forward signaling promoted human mesenchymal stem cell migration [29]. On the other hand, it has been reported that ephrin B1/EphB3 mediated forward signaling acts as a mitogen to regulate palatal shelf mesenchymal cell proliferation via activation of the ERK/MAPK signaling pathway [27]. In the mouse small intestine and colon, EphB2 signaling directs stem cell migration, promotes cell-cycle reentry of progenitor cells and stimulates cell proliferation [22]. Accordingly, these studies indicate that functions of ephrin/Eph forward signaling are complex and depend on the target tissue and cell-types. The relative contribution of eprhin/Eph forward signaling in skull development, bone formation and bone resorption is not well understood. In our studies, we did not observe impaired bone formation in mice with disruption of ephrin B1 gene in myeloid lineage cells. Instead, we found a slight, but not significant increase in bone formation. The slight increase in bone formation could be a compensatory effect to increased bone resorption. In addition, osteoblasts and mesenchymal stem cells express abundant eprhin B1 protein which can compensate for the loss of ephrin B1 produced in osteoclasts, and interact with its receptors on the membrane of neighboring cells. Thus, our observation that trabecular bone volume was diminished in the ephrin B1 conditional KO mice cannot be explained because of lack of receptor-mediated forward signaling in the bone.

Rapid cytoskeletal reorganization is essential for osteoclast function and formation of the specialized membranes. Recent studies have found that NHERF1 expressed in osteoclasts co-localizes with type IIa Na/Pi contransporter (Npt2a) and actin at the plasma membrane and associates with ezrin and actin for membrane sorting [21]. Targeted disruption of NHERF1 results in postnatal lethality often accompanied by bone fractures due to 25–30% reduction in bone mineral density [30] while targeted disruption of Npt2a only showed mild skeletal phenotype that is improved with age [31]. The severity of skeletal phenotype in the NHERF1 KO mice cannot be explained by the sole function of NHERF1 to redistribute Npt2a for phosphate transport in the kidneys, thus suggesting that NHERF1 has other functions such as modulation of osteoclast differentiation and bone resorption [21]. Moreover, interaction of NHERF1 with PDGFR-β through its C-terminal PDZ binding motif modulates PDGF-induced cell spreading and motility [32], [33], and the formation of a ternary complex between PTEN, NHERF1 and PDGFR have been reported to attenuate PDGF-induced cytoskeletal rearrangements and chemotactic migration of the cells [34]. In terms of the molecular pathway of ephrin B1 in bone cells, we previously demonstrated that ephrin B1 interacts with PTPN13 and NHEFR1 in bone marrow stromal cells [16]. Here, we show that ephrin B1, which is structurally similar to PTEN, also interacts with NHERF1 in osteoclasts, and treatment of osteoclast with EphB2-Fc leads to reduced phosphorylation of ERM proteins. Because NHERF1 can interact with both cytoplasmic PDZ domain of membrane proteins and N-terminus of ezrin [35], it is reasonable to assume that the interaction of ephrin B1 with NHERF1 could disrupt the intramolecular interaction of “head-to-tail” of NHERF1, and stabilize it in an “open” conformation so that C-terminal ERM binding motif of NHERF1 can associate with ERM with high affinity at the osteoclast membrane [36]. Studies in endothelial cells have also revealed that C-terminal PDZ motif of G protein coupled receptors and membrane iron channels can recruit PDZ domain-containing protein phosphatase PP2A and NHERF1 at the apical surface to form scaffold protein complex [35], [37], [38]. Therefore, it is possible that complex formation of ephrin B1, NHERF1 and ERM facilitates ezrin dephosphorylation via PP2A and modulates the lateral mobility of osteoclast precursors. Furthermore, clustered ephrin B1 polymers, NHERF1, Npt2a, and/or Na^+^/H^+^ exchanger 3 may form scaffold complex for regulation of membrane sorting, apical membrane mobility and Na^+^/H^+^/Pi transport in osteoclasts when osteoclasts contact osteoblasts on the bone surface [21], [34], [35]. In support of these hypotheses, our experiments show that the ERM inactivation is associated with reduced osteoclast size, and resorptive activity. Activation of ephrin B1 reverse signaling also inhibited TRAP, cathepsin K and NFATc1 expression. Our data suggest that besides ephrin B1 reverse signaling regulates the expression of NFATc1, it also modulates phosphorylation of ERM proteins which have been shown to be involved in the rearrangement of actin cytoskeleton in neuronal morphogenesis [20]. Further studies are needed to determine whether activation of ephrin B1 reverse signaling regulates osteclast differentiation and bone resorption via rearranging cytoskeleton, and whether PDZ domain containing phosphatase such as PP2A is involved in the ephrin B1/NHERF1 complex formation and mediates ERM dephosphorylation.

## Materials and Methods

### Recombinant proteins and antibodies

Recombinant proteins of control Fc and EphB2-Fc, anti-human IgG, anti-NHERF1 and anti-β-actin were purchased from Sigma (St. Louis, CO). Recombinant proteins of M-CSF and RANKL, anti-ephrin B1, anti-ephrin B2, anti-RANK and anti-M-CSR were from R & D Systems (Minneapolis, MN). Anti-ezrin, anti-ezrin/radixin/moesin and anti-phospho-ezrin (Thr567)/radixin (Thr564)/moesin (Thr558) were products of Cell Signaling Technology (Danvers, MA).

### Generation of mutant mice

The floxed ephrin B1 mice with mixed background of C57BL/6J and 129S4 strains were kindly provided by Dr. Philippe Soriano [1], [5]. The Lyz2-Cre knock-in mice expressing Cre recombinase (Cre) were purchased from The Jackson Laboratory (Bar Harbor, Main) [39]. To generate conditional KO mice that lack ephrin B1 in cells of myeloid lineage, *efnb1* ^flox/flox^ female was first crossed with LyZ2^Cre/Cre^ male to generate LyZ2^Cre/Wt^/Efnb1^loxp/wt^ females. LyZ2^Cre/Wt^/Efnb1^loxp/wt^ females were then bred with *efnb1* ^flox/Y^ hemizygous males to generate LyZ2^Cre/Wt^/Efnb1^loxp/loxp^ females and with LyZ2^Cre/Wt^/Efnb1^loxp/Y^ males. In subsequent breeding, LyZ2^Cre/Wt^/Efnb1^loxp/loxp^ females were crossed with LyZ2^Cre/Wt^/Efnb1^loxp/Y^ males. Our breeding strategy should yield 25% of LyZ2^Cre/Cre^/Efnb1^loxp/Y^ males and LyZ2^Cre/Cre^/Efnb1^loxp/loxp^ females (KO), 50% of LyZ2^Cre/Wt^/Efnb1^loxp/Y^ males and LyZ2^Cre/Wt^/Efnb1^loxp/loxp^ females (Heterozygous Cre), and 25% of LyZ2^Wt/Wt^/Efnb1^loxp/Y^ males and LyZ2^Wt/Wt^/Efnb1^loxp/loxp^ females (WT) mice. We excluded Lyz2-Cre heterozygous mice for experiments because Cre expression in these mice may be low and insufficient to delete ephrin B1 gene. Genotyping of the ephrin B1 gene and LyZ2 locus were monitored by PCR using DNA extracted from tail snips as reported [5], [16], [39]. All mice were housed at the Jerry L. Pettis Memorial VA Medical Center Veterinary Medical Unit (Loma Linda, CA) under standard approved laboratory conditions with controlled illumination (14 hours light, 10 hours dark), temperature (22°C) and unrestricted food and water. All of the procedures were performed with the approval of the Institutional Animal Care and Use Committee (IACUC) of the Jerry L Pettis Memorial VA Medical Center.

### Evaluation of bone phenotypes

Micro-architectures of the femurs isolated from 21-week old mice were assessed by μ-CT (Scanco Invivo CT40, Switzerland) as described previously [16], [40]. Routine calibration was performed once per week using a three-point calibration phantom corresponding to the density range from air to cortical bone. The femurs were fixed in 10% formalin overnight, washed with PBS and immersed in PBS to prevent them from drying. The bone was scanned by X-ray at 55 kVp volts (trabecular bone) or at 75Kvp volts (cortical bone) at a resolution of 10.5 µm/slice. To minimize the position error (slice positioning) and to be consistent in our sampling site from mouse to mouse, we undertook several precautionary steps, which include: 1) the use of scout view of the whole femur to determine landmarks and precise selections of measurement sites; 2) the use of the growth plate of the distal femurs as the reference point; 3) use of a 0.525 mm sampling site that represented 0.315 mm away from the growth plate for measurement of trabecular bone parameters, and 4) the use of a 1.05 mm sampling site that represented 5.5 mm away from growth plate for measurement of cortical bone parameters. After acquiring the radiographic data, images were reconstructed by using 2-D image software provided by Scanco. The area of the trabecular analysis was outlined within the trabecular compartment. Every 10 sections were outlined, and the intermediate sections were interpolated with the contouring algorithm to create a volume of interest, followed by 3-D analyses using Scanco in vivo software. Parameters such as bone volume (BV, mm^3^), bone volume fraction (BV/TV, %), trabecular number (Tb.N, mm−^1^), trabecular thickness (Tb.Th, µm) and trabecular separation (Tb.Sp, µm) were evaluated. The bones analyzed were adjusted for length so that the regions of interest chosen for cortical and trabecular bone parameters were anatomically the same if there is a difference in bone length between the mutant mice and control littermates.

### Dynamic calcein labeling and histomorphometry

Twenty-one week-old mice were injected intraperitoneally with calcein eight days (20 mg/kg) and two days prior to the expected day of euthanization in order to label mineralizing bone surface. Mouse femurs were fixed in 10% formalin overnight. The bones were washed, dehydrated, embedded in methyl methacrylate resin for sectioning. Longitudinal sections of comparable anatomic position of the femurs were analyzed by fluorescence microscopy. For analysis of trabecular bone formation parameters, distal metaphysis of left femurs were used as a sampling site. For evaluation of bone resorption parameters, the right femurs were partially demineralized, embedded in glycomethacrylate and cut into sections. Seven microscope fields per bone section that cover all trabecular bones of the metaphyseal region of the distal femur were used for the TRAP surface measurement. We excluded cortical bone and the trabecular bone that is adjacent to growth plate since active remodeling is taking place at this place. The postcoupling method with Napthol-AS-BI phosphate was used as the substrate and diazotized pararosaniline was served as the coupling reagent to stain for TRAP activity. Two middle longitudinal sections per animal were stained and counted. The trabecular surface and the TRAP labeled trabecular surface were measured in a blinded fashion with computer software OsteoMeasure (Osteometrics, Inc. Decatur, GA) by our histomorphological core facility [41], [42]. Mineral apposition rate, bone formation rate/bone surface were calculated as described previously [43].

### Serum TRAP activity assay

Mouse sera were collected from 21-week old mice as described above. Total serum TRAP activity was measured as previously reported [44].

### *In vitro* osteoclast formation

Primary osteoclast precursors were isolated from the spleen or bone marrow of long bones (femur and tibia) of 5-week old ephrin B1 conditional KO mice and corresponding control littermates as described previously [45]. The isolated precursors are maintained in α-MEM supplemented with 10% fetal bovine serum (FBS), penicillin (100 units/ml), streptomycin (100 µg/ml), and macrophage colony stimulating factor (M-CSF) (20 ng/ml) at 37°C in 5% CO_2_ for 2 days to stimulate monocyte proliferation. To induce osteoclast differentiation, trypsinized precursors were seeded to 96-well plates (2500 cells/well) or 48-well plates (5000 cells/well), and incubated with M-CSF (20 ng/ml) and RANKL (30 ng/ml). The medium was changed every 2 days. Osteoclastogenesis was evaluated by counting TRAP staining positive, multinucleated cells with more than three nuclei after TRAP staining (Sigma Aldrich, St. Louis, MO).

### RNA extraction and quantitative PCR

RNA was extracted from primary osteoclast cultures as described previously [40], [45]. An aliquot of RNA (2 µg) was reverse-transcribed into cDNA in 20 µl volume of reaction by oligo(dT)_12–18_ primer. Real time PCR contained 0.5 µl template cDNA, 1× SYBR GREEN master mix (ABI), and 100 nM of specific forward and reverse primers in 25 µl volume of reaction. Primers for peptidyl prolyl isomerase A (PPIA) were used to normalize the expression data of interest genes. Sequences of the primers used are listed in Table 1. PCR amplification efficiencies with specific primers to mouse ephrin B1 and ephrin B2 were calibrated using different copy numbers of plasmids of pMX-ephrin B2 and pMX-ephrin B1. In repeat real time PCR using known copy numbers of ephrin B1 and ephrin B2 plasmids, the difference in PCR amplification efficiency for the two sets of ephrin B1 and ephrin B2 primers was within 10% with almost same delta CT.

**Table 1:** Primer sequences for real time RT-PCR.

Gene	Forward	Reverse
Ephrin B1	5′-TGCAACAAGCCACACCAGGA	5′-CGACGGCTGCGAACAATGCT
Ephrin B2	5′-AGCCCTAACCTCTGGGGTCT	5′-GCCATCGGTGCTAGAACCTG
Cathepsin K	5′-GAACGAGAAAGCCCTGAAGAGA	5′-TATCGAGTGCTTGCTTCCCTTC
NFATc1	5′-ATACTTCCTGTCCTCTGGCAACA	5′-GCTTGCAGCTAGGAAGTACGTCTT
PPIA	5′-CCATGGCAAATGCTGGACCA	5′-TCCTGGACCCAAAACGCTCC
TRAP	5′-CACTCAGCTGTCCTGGCTCAA	5′- CTGCAGGTTGTGGTCATGTCC

### Bone resorption pit assay

Slices from bovine cortical bone were placed in the bottom of 24-wells and cells were seeded on top of bone slices. Cells on bone slices were removed in 10% sodium hypochlorite. Air dried bone slices were stained with hematoxylin. The entire surface of each bone slice was examined and the total resorbed area per bone slice was quantified using ImageJ (National Institutes of Health).

### Immunoprecipitation

Cultured osteoclasts were lysed with lysis buffer (50 mM HEPES [pH 7.5], 100 mM NaCl, 10 mM EDTA, 10% glycerol, 1% Triton X-100, 1 mM phenylmethylsulfonyl fluoride, 1× protease inhibitor cocktail and 1× phosphatase inhibitor cocktail). Cell lysate (300 µg of total protein) was first precleared by using protein A/G-Sepharose and then incubated with 4 µg of first, antibody specific to NHERF1 or control IgG for 1 hour at 4°C with gentle shaking, followed by the addition of protein A/G-Sepharose and an additional overnight incubation at 4°C. After centrifugation, the protein A/G-Sepharose beads were washed five times with cold lysis buffer and then boiled with SDS-PAGE sample buffer to dissociate the proteins. The immunoprecipitated proteins were separated by SDS-PAGE under reducing conditions for Western Blot with antibodies against ephrin B1 [16].

### Western blot analyses

Cultured osteoclasts were lysed as described above. Cell lysate (30 µg of total cellular protein) was separated by 8–10% SDS-PAGE under reducing conditions for Western Blot with specific antibodies against ephrin B1, ezrins, and β-actin as described previously [46].

### Statistical analysis

Data were analyzed using Student's T-test or ANOVA (one-way or two way) (Statistica 6, Tulsa, OK) as appropriate.
